# Looking through the lens: a photovoice study examining access to services for newcomer children

**DOI:** 10.1080/17482631.2023.2255176

**Published:** 2023-09-08

**Authors:** Nahal Fakhari, Jessie-Lee D. McIsaac, Rebecca Feicht, Sarah Reddington, Susan Brigham, April Mandrona, Christine McLean, Mary Jane Harkins, Emma Stirling Cameron

**Affiliations:** a Early Childhood Collaborative Research Centre, Mount Saint Vincent University, Halifax, Canada; b School of Health and Human Performance, Dalhousie University, Halifax, Canada; c Faculty of Education, Mount Saint Vincent University, Halifax, Canada; d Department of Child and Youth Study, Mount Saint Vincent University, Halifax, Canada; e Division of Art History and Contemporary Culture, NSCAD University, Halifax, Canada; f Faculty of Medicine, University of British Columbia, Vancouver, Canada

**Keywords:** Newcomer families, newcomer children, early childhood educators, early childhood programs, culturally responsive, photovoice

## Abstract

**Purpose:**

Canadian new immigrant families (also known as newcomers) encounter challenges navigating systems when trying to access programmes critical for their children’s healthy development. The purpose of this study is to understand how newcomer families find and use early childhood programmes and services from the perspective of families and early childhood educators (ECEs) working within a settlement organization.

**Methods:**

Using photovoice methodology, newcomer family members (*n* = 8) with young children and ECEs (*n* = 6) participated in a series of virtual workshops to share photos and reflect on their experiences.

**Results:**

Participants discussed the systemic barriers that obstructed newcomer families’ access to services for young newcomer children. Financial challenges due to unemployment/underemployment, language and cultural differences were emphasized. Despite these barriers and challenges, participants shared how culturally responsive programmes enhanced their connections to programmes and services. Both groups of participants discussed the critical role of social networks in supporting newcomers to use programmes by helping families become aware of available services and assistance with various processes such as registration.

**Conclusions:**

This research illustrates the lived experiences of newcomer families and identifies opportunities to address inequities, improve early childhood programmes, and enhance families’ access to programmes and services.

## Introduction

In recent years, communities in the east coast province of Nova Scotia have become increasingly diverse; the population has recently exceeded one million people, largely as a result of immigration (Nova Scotia, 2021). However, communities in Nova Scotia have fewer resources to support settlement compared to large Canadian cities and are less diverse, especially considering its smaller population in contrast to metropolitan areas (Government of Canada, S. C, 2017). This increase in population in Nova Scotia underscores the importance of providing relevant supports and services for *newcomers* (individuals who fall within Canada’s immigration categories, including economic immigrant, sponsored immigrant by family, refugee, temporary worker, and undocumented) (Thevenot, 2021). The current gap in support is evident when reviewing studies of newcomer experiences where families’ resettlement experiences are often accompanied by adjustments and challenges as they learn to understand, manage, and navigate new physical, social, and cultural settings (Brown et al., 2020, Khanlou, 2010, Khanlou & Crawford, 2006). Newcomer families with young children may require more individualized, person-centred supports, and therefore, research is needed to better understand their lived experiences and identify strategies to support the well-being and learning of newcomer children (Karoly & Gonzalez, 2011, McHugh & Margie, 2014).

### Background

Newcomer families experience significant barriers in navigating Canadian systems and challenges (Brosinsky et al., 2018, Brown et al., 2020). A recent scoping review highlights themes relating to intercultural responsiveness and systemic barriers connected to immigration status that impact newcomer family experiences with early childhood programmes and services (Brown et al., 2020). Key areas emerging from this scoping review identified post-migration systemic barriers that limit access to programmes, such as language, employment, and education. Psychosocial factors were also identified as influencing newcomer families’ experiences, including individual perceptions and broader social supports and networks (Brown et al., 2020). Indeed, community assets across newcomer networks have been shown across multiple studies as critical in improving meaningful access to programmes and services, and thereby, newcomer experiences (Ansion & Merali, 2018, Klassen et al., 2012, Wahoush, 2009). Through studying experiences of newcomer families, past research suggests the affordability and accessibility of programmes are important to newcomer populations as well as consideration of cultural practices and language needs (Brown et al., 2020, Choi et al., 2014, Leung et al., 2019, Massing, 2021, Salami et al., 2019, Salami et al., 2020, Salami et al., 2022).

The COVID-19 pandemic and resultant public health restrictions have disrupted early childhood programmes and services across Canada and further complicated access to and experiences of services for newcomer families. Recent research describes specific experiences of newcomer families that prevent inclusive and equitable access to language and employment programmes and health services. For example, when programmes shifted to virtual platforms, supports and services for families were sometimes limited due to technological barriers and limited resources (e.g., interpreters, language services) (Benjamen et al., 2021, Etowa et al., 2021, Stirling Cameron et al., 2021). Guruge et al. (2021) further found that in the metropolitan city of Toronto (Canada), newcomer caregivers with early English proficiency felt inadequate and alone in supporting their children’s education through virtual platforms (Guruge et al., 2021).

Social support is critically important for connecting with community programmes, but the isolation and lockdown that was experienced throughout COVID-19 has left families with minimal support (Stirling Cameron et al., 2021). Similarly, other researchers found that during the pandemic newcomers were more concerned about losing their social networks than Canadian-born populations (LaRochelle-Côté & Uppal, n.d..). As newcomer families typically experience disparity that impacts their capacity to gain access to services and community programmes, due to language, cultural, and financial challenges (Brosinsky et al., 2018; Browne et al., 2017, Browne et al., 2018), it is essential to understand and address how the pandemic exacerbated these difficulties.

The Nova Scotia Department of Education and Early Childhood Development (DEECD) identifies early childhood educators (ECE) as skilled professionals who provide quality and inclusive early learning and child care children and families. ECEs are recognized as researchers and collaborators that provide learning environments that are holistic, inclusive and reflect children’s interests, ideas and curiosities and the broader context of their families, communities, language, and culture (Nova Scotia, 2023). Strong partnerships between families and ECEs can enrich a child’s early learning experience when families are invited to contribute their ideas, abilities, knowledge, and experiences (Perlman & Fletcher, 2012, Reynolds & Duff, 2016). In addition to their role in formal child care, educational, and settlement settings, ECEs provide informational support and connect newcomer families to organizations like schools, social welfare programmes, community programmes, maternal education, and employment (Vesely et al., 2013). Importantly, ECEs who are also newcomers and represent families’ cultural and language backgrounds, have in depth understanding of newcomer families’ experiences after migration (Massing, 2021). As a result of these experiences, ECEs working with newcomer families offer an important perspective on family experiences (Massing, 2021, Pacini-Ketchabaw & Bernhard, 2018).

### Theoretical framework: a feminist post-structural approach

Migration is a complex process of social, emotional, political, and environmental factors that impact new immigrant families’ decisions to move to cities, such as Halifax, the capital city of Nova Scotia. It brings new realities and challenges for families with diverse lived experiences. In this study, an interdisciplinary feminist post-structural lens is used to better understand issues of marginalization, including how gender impacts migration for newcomers to Nova Scotia. Gender as a social construct traditionally informs the organizing structures of families (e.g., gender roles, embedded family power structures) and therefore, gender influences how immigrant families’ navigate Canadian systems (Azzarito et al., 2006, Hesse-Biber, 2012, Weedon, 1987). Prior research has reported on the gendered realities of the immigration experience, particularly as it relates to caregiving for young children. For example, it is found in Toronto that traditional gender roles like child rearing responsibilities influenced Chinese mothers’ employment seeking behaviour (Leung et al., 2019). Another study conducted in Nova Scotia demonstrated that Syrian immigrant mothers who had the main role in taking care of their children had limited ability to attend their health care appointments due to the absence of affordable child care (Stirling Cameron et al., 2022). Further, hooks (1994) explains that we need to theorize from the struggles and pain that individuals embody and that we do this from a place where we move to “recover and remember ourselves” (p. 74).

A feminist post-structural approach to this research acknowledges that gender is an inflection of how social relations, community intersections, and structures of power in and outside the family system impact newcomer identities, with recognition of subjectivity, in particular recognizing the resilience, coping, and learning of newcomers alongside of the challenges they face (Barrett, 2010, Hesse-Biber, 2012, Weedon, 1987). This conceptual framework attends to variations in experience and offers a distinct ontological stance to consider the transformative aspects of lived experience (Lather, 1997). In this research, a feminist post-structural lens provides opportunity for newcomers and ECEs to share their stories contextualize the complex dynamics when navigating their roles and familial expectations as new immigrants to Halifax, Nova Scotia. Here, the mobilization of a feminist post-structural lens is intended to open up new ways of thinking about social relations, gendered realities, community intersections, and structures of power in and outside the family systems that impact the lived experiences of newcomer families in Nova Scotia as they access supports and services. This critical feminist stance allows us to move beyond conventional forms of representation with an emphasis on the first voice perspectives of newcomers when navigating services in Nova Scotia (Azzarito, 2012; Lather, 1997; St. Pierre et al., 1999; Weedon, 1987).

### Purpose

The purpose of this study is to understand how newcomer families find and use early childhood programmes and services from the perspective of families and ECEs working within a settlement organization. Eight Arabic-speaking newcomer family members with the support of an Arabic interpreter and six ECEs participated in this project. We aimed to achieve the following objectives, which are aligned with photovoice methodology (Wang & Burris, 1997):

1. To enable immigrant families and ECEs to record and reflect on the assets and concerns;
2. To promote critical dialogue, giving voice to immigrant families’ and ECEs’ experiences and concerns through visual methods; and
3. To facilitate knowledge mobilization with information and evidence relevant to the experiences and understandings of immigrant families and ECEs.

## Methods

### Photovoice methodology

The use of photovoice as our methodology supports this inquiry as it is shaped by feminist theory through its prioritization of people who have been systematically excluded from traditional research (Wang & Burris, 1994). Photography has the power to affect emotions and communicate feelings, ideas, and experiences (Brigham et al., 2018, 2018), which makes it a compelling medium for research. Photovoice, which was originally developed by Wang and Burris in 1994, is “a collaborative research method in which research participants are actively involved in taking photographs to document their lived experiences, tell their stories, explore community needs, and create awareness of their experiences and circumstances within a group, and possibly with a wider audience” (Brigham & Kharbach, 2020, p. 156). Photovoice aims to shift policy and ignite community change through destabilizing universal notions of experience and mobilizing new knowledges from marginalized populations through co-creation, reflection, and critical group dialogue (Strack et al., 2004, Wang & Burris, 1997).

Researchers have explored a range of issues using photovoice related to health, education, and other social issues with a variety of underrepresented population groups, including immigrants (Brotman et al., 2017, Haque & Eng, 2011, Rania et al., 2015), and refugee and immigrant youth and women (Brigham, 2015; Brigham et al., 2018; Brigham et al., 2018; Fassetta, 2016; Robertson et al., 2016). In addition to the strengths of the methodology described above, photovoice was appropriate for our study population as English was not the first language of the newcomer participants, so photographs allowed for sharing stories without relying on the spoken or written word (Drolet et al., 2008; Miller, ,^2021^; Wang et al., 2006; Sethi, 2016; Strack et al., 2004; Sutherland & Cheng, 2009; Wang & Burris, 1997).

We used the 9-step approach described by Wang (2006) as outlined in Table I. As this research was conducted during the COVID-19 pandemic, the photovoice process was adapted for Microsoft Teams and Zoom. With the permission of our participants, all the sessions were audio and video-recorded. We acknowledge the inherent limitations of virtual data collection and will later discuss the associated implications. The ECE participants participated in six virtual workshops each lasting around two hours between April and May 2021; the family group participated in four virtual workshops each lasting around one hour between July and October of 2021.

**Table I.** Photovoice steps employed with participant groups.

Photovoice methodology steps	ECE Participants (April – May 2021)	Family Participants (July- October 2021)
Steps 1 & 2: Connecting with community and recruiting participants	This research was co-developed with partners at a local settlement agency who also supported the recruitment process.	
Steps 3 & 4: Introducing the project to interested participants and establishing consent	Interested participants voluntarily participated in a virtual meeting with the research coordinator to establish informed consent.	
Steps 5 & 6: Brainstorming ideas for photo taking, photography training	Virtual workshop 1: Review of project details, discussion of ideas for photos, photography training.	
Step 7: Time for photo taking	Participants were invited to spend 1–2 weeks taking photos based on guiding question.	
Step 8: Data management and analysis (Selecting, contextualizing and codifying using SHOWeD* method)	Virtual workshops 2, 3, 4: selecting and contextualizing individual photos through discussion (invited to take additional photos between workshops to build discussion).	Virtual workshops 2 & 3: selecting and contextualizing individual photos through discussion (invited to take additional photos between workshops to build discussion).
	Virtual workshops 5 & 6: contextualizing and codifying photos using virtual brainstorm platform.	Virtual workshops 4 & 5: contextualizing and codifying photos using virtual brainstorm platform.
Step 9: Knowledge mobilization	Participants discussed their ideas for knowledge mobilization, including an outdoor photo exhibit at a public space.	

NOTE: * Discussion using SHOWeD method: See, what they think is really happening, How what is happening relates to Our lives, Why the issue/problem/strength exists, and what can be Done about it (Wang & Burris, 1994, Wang & Burris, 1997).

### Recruitment, participants, and informed consent

This project received research ethics approval from the relevant university review boards. We partnered with the local settlement agency, which provides services and supports to newcomers in the capital city of Halifax (Nova Scotia) to enable recruitment and support the research process. All newcomer families who had used the child care programme with our settlement partner in the past five years were eligible to participate in the study. The recruitment posters were translated in Arabic based on the recommendation of our settlement partner and both English and Arabic posters were distributed within the settlement agency and broader community.

Prior to the first workshop, an information package was emailed to potential participants. Materials sent to the participants were translated into Arabic (as this was the first language of all the newcomer family participants) to support participants’ understanding of the study and informed consent process. As taking photographs is a political act (Castleden et al., 2008), the team spent time discussing the ethics of photography and informed consent with participants before and during the research process. This included a virtual meeting with the research coordinator that reviewed study information, informed consent for the study, permission to take photos of others, and use photos for research dissemination. Consent was obtained verbally from participants prior to the first workshop and reviewed at subsequent sessions. The researchers have securely stored records of the participants’ consent to use their photos in this paper.

Eight newcomer family members living in Nova Scotia between 1.5 and 13 years participated in this research (see Table II). All participants had refugee backgrounds, were Arabic speaking and identified their countries of origin as Iraq (5), Syria (2), and Sudan (1). An Arabic-speaking interpreter accompanied the five newcomer families (six mothers and two fathers) who required an interpreter for active participation in the study and supported them in reviewing the information package and establishing consent. In one case, a participant relied on another participant who is a relative and has proficiency in English to interpret the conversation.

**Table II.** Participant characteristics.

Family Participants					
Participant Pseudonym	Research grouping	Country of origin	First language	Number of children	Number of years since migration
Amal	Individual	Sudan	Arabic	2	2
Ahlam and Abbas	In pair with spouse	Iraq		1	3.5
Afra and Akbar	In pair with spouse	Iraq		3	3
Amira	Individual	Iraq		4	13
Annisa	In pair with sibling	Syria		2	1.5
Habiba				6	5
ECE Participants					
Participant Pseudonym	Occupation in home country	Country of origin	First language	Number of children	Number of years since migration
Charlize	Social worker	South Africa	Afrikaans/ English	3	2.5
Charuka	Teacher	Sri Lanka	Sinhala	1	2.5
Fatima	Social worker	India	English/Urdu	1	2.5
Juliana	Business owner	Brazil	Portuguese	3	5
Charlotte	ECE	Canada	English	1	7 years in Nova Scotia
Yao	Teacher	China	Mandarin	2	5

For our ECE group, the process of recruitment was targeted by inviting any educator working with the settlement agency to participate. The consent and research processes were similar to that our newcomer families as described above. Although not an eligibility requirement, five out of six ECE participants were newcomers who came to Canada between 2 and 5 years ago and had not previously been employed in the early childhood education field before immigration. All ECEs identified as mothers and shared their own personal experience as well as the perspective of a professional working with newcomer families.

### Procedures

#### Data collection

Newcomer participants were given the option to attend the virtual workshop individually, with a family member, or as a larger group with other participants. The ECEs had an existing relationship with one another and similar schedules, which allowed the workshop to be conducted as a group. All workshops were audio-recorded and transcribed verbatim.

The initial workshop focused on reviewing the project details, establishing rapport among participants and with the research team, and brainstorming ideas for photos that could address the guiding question: *How do newcomer families find and use programmes and services for their young children?* A brief photography training session was provided, and participants were invited to spend 1–2 weeks taking photos. Participants could choose to take photos with their own devices (such as cell phones), or they could borrow a digital single-lens reflex camera from the research team. They were encouraged to document a rationale for why they took their photographs and a brief description of the photos using a worksheet provided in their information package (e.g., *Why did you take this photo? What do you want people to understand when they look at this photo?)*. Subsequent workshops (see Table I) were held to discuss the photos and their meanings. Participants used a variety of methods to send their photos to the research team (e.g., text message, email, Microsoft OneDrive folder). The participants were advised to number their photos based on their significance. The research team uploaded the photos using the participant-determined order on a virtual brainstorming platform, Google Jamboard, which was then screen shared by the research team during the analysis workshop. The first two or three photos from each participant were shared during the discussion sessions.

#### Data analysis

Data analysis followed the three-step photovoice analysis process that includes selecting, contextualizing, and codifying (Wang & Burris, 1997, Wang, 2006, Wang et al., 1998). We began this process by asking each participant to select photos they felt to be meaningful, important, or significant to share with the group. Contextualization occurred by inviting the participant who took the photo to share their thoughts and feelings relating to the photo and then other participants were invited to contribute their ideas, ask questions or make comments. Contextualization was furthered through use of the SHOWeD acronym as guiding questions (Table I), which was used by the researchers to prompt discussion related to the meaning of the photos (Wang & Burris, 1994, Wang & Burris, 1997, Wang, 2006). The SHOWeD method asks participants what they see, what they think is really happening, how what is happening relates to our lives, why the issue/problem/strength exists, and what can be done about it (Wang & Burris, 1997). A member of the research team used virtual notes on Google Jamboard to record participants’ comments related to the photos. Participants were encouraged to contribute their ideas directly on Google Jamboard or to verbally convey their ideas to the research team who added them to Google Jamboard.

Finally, participants were engaged in codifying, or a form of participatory thematic analysis that invites participants to examine commonalities, themes, patterns, or trends across photos (Wang et al., 1998). At the final workshop, participants were asked to group together photos, ideas, quotes, and Jamboard notes to identify the main findings reflected across all workshops (Wang & Burris, 1997, Wang, 2006). For the newcomer family participants, a separate schematic was developed in discussion with each participant (or pairing). The research team developed a collective schematic to reflect ideas across the newcomer family participants. Then, the newcomer family participants were invited to take part in a final workshop as a group to further refine the schematic (Figure 3). For the ECE group, a visual schematic was also developed through discussion to reflect the interconnection amongst participants’ ideas. An initial model was collaboratively developed during the workshop, refined by the research team, and confirmed by the ECE participants in a final workshop (Figure 4).

## Results

**Figure 1.**
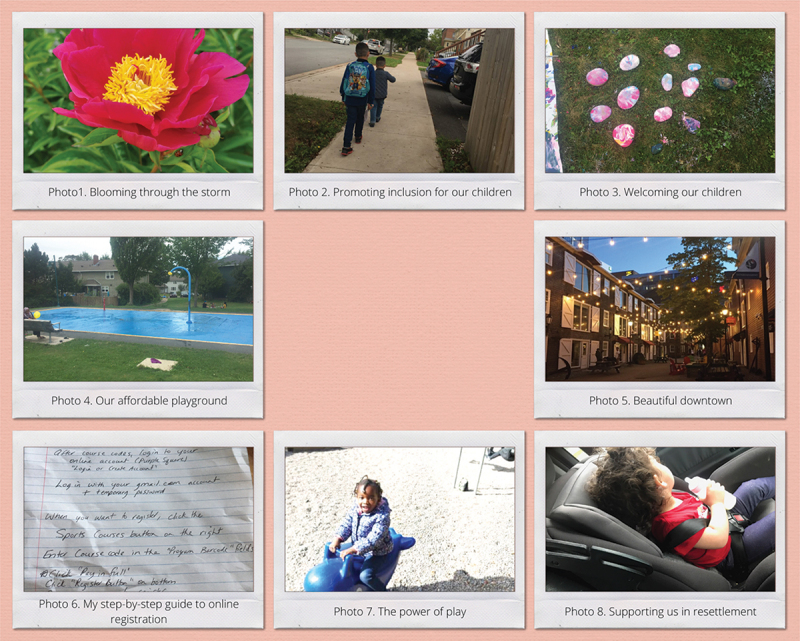
Family participants’ photos.

**Figure 2.**
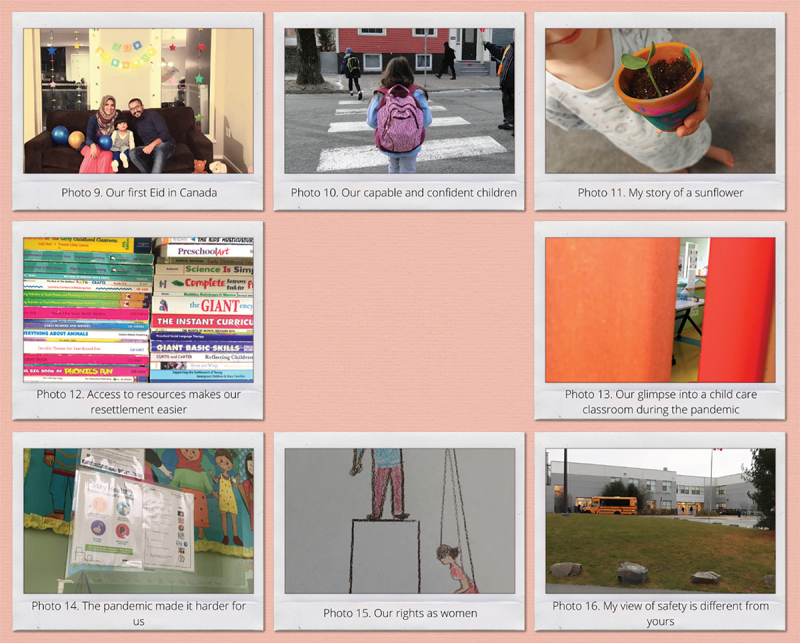
ECE participants’ photos.

This section describes two separate sets of themes that were developed by the newcomer families and ECEs during their workshops. The ECEs subsequently developed four themes highlighting their perceptions of families attending their programme, including their own experiences as newcomers. This section presents the participants’ quotes alongside of the photos that they took. Participants’ photos are presented in two separate collages in Figures 1 and 2.

### Newcomer families

**Figure 3.**
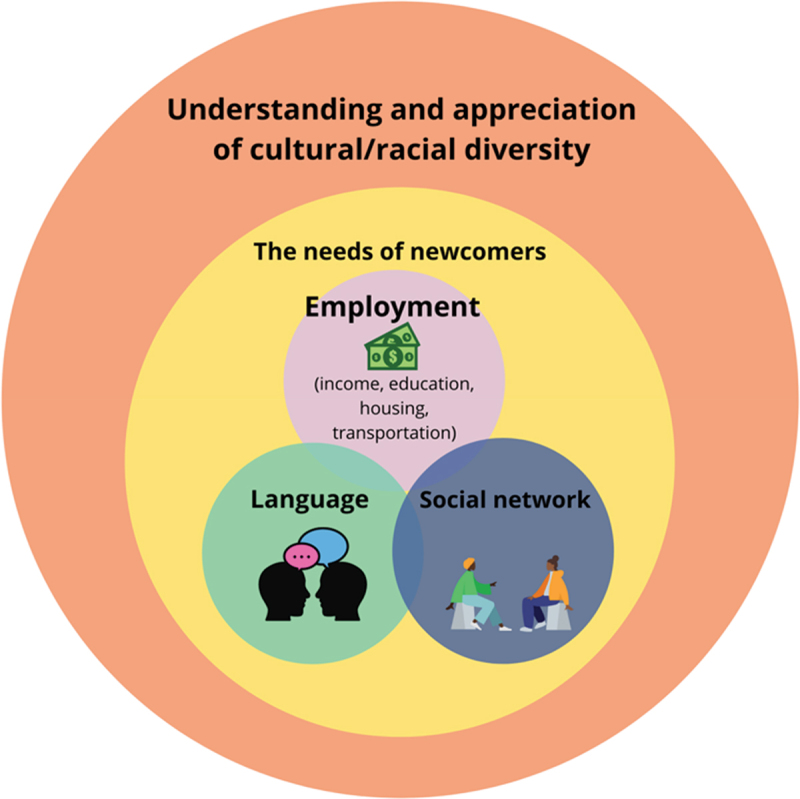
Visual representation of newcomer families’ results.

Figure 3 illustrates how newcomer families describe their experiences finding and using programmes for their children. Their experiences were influenced by the understanding and appreciation of cultural/racial diversity in the broader community, along with consideration of the unique needs of newcomer families, especially with respect to language, employment, and social networks.

#### Understanding and appreciation of cultural/racial diversity

Family participants were more willing to use programmes and services that respect their *cultural and racial diversity* and demonstrate an understanding of newcomer post-migration challenges. All family participants indicated that their children did not always feel welcome at school, especially upon arrival, due to linguistic barriers and the ignorance of dietary, religious, and cultural differences that became obvious during important events such as Ramadan. For example, children that did not have an English language background could not as easily communicate with peers and teachers, and therefore felt frustrated, upset, and excluded. For example, Ahlam felt disrespected and unwanted when her child was bullied at school or when she heard of attacks towards Muslim communities in another province in Canada.:

*“The bullying is you know very important issue and actually we suffer here too much from it you know … you know some people use this point against him they tell him ‘you are weak’, like this is you know they put some word and he have some bullying …* . *Nobody listen to him or me, there is no teacher or person who will listen to us. This is a really big problem if he lost trust in the school … .”*

Family participants described how they navigated these post-migration experiences to find support and services that would help them, such as Ahlam who participated in mental health programmes to gain more knowledge on how to maintain a positive attitude as her family navigated post-migration stress and discrimination. Ahlam took a picture of a blooming flower in front of a rock to represent how hopeful she is for her family’s future (Photo 1).

While recognizing the challenges, other examples were shared from participants about the ways that services were inclusive to families. Annisa took a photo of her children attending school, expressing her appreciation for a school system that did not exclude children with diverse learning needs, such as her son with autism (Photo 2):

*I’m so glad for my son, he’s now having friends. For example, when he leaves school his friends say bye to him. They know that he doesn’t speak but […] they say bye to him. In class, teachers tells his friends to sit next to him, to read stories for him.*

Family participants also spoke about how their immigration status sometimes prevented them from using programmes. Children’s activities offered by settlement agencies are one of the main programmes accessed by newcomer families; however, these programmes are mainly offered to families with permanent residence status. Amira, who struggled to find free of charge programmes for her children, shared a photo of a free outdoor activity that her children attended during summer and appreciated that her children were not excluded because of their citizenship status (Photo 3) saying: “ *… I have four kids. What’s different between the people? … The people [that] have citizenship, and the people doesn’t have [citizenship], you know here everything expensive.”*

#### The needs of newcomers

Family participants identified the fundamental needs that impact their access to programmes for their children, including employment, language support, and help from their social network.

##### Employment

Lack of access to employment opportunities was identified as one of the main concerns of newcomer families to access programmes and services for their children. Most participants faced challenges finding employment due to a lack of recognition of their education and qualifications and/or language barriers. Insufficient income meant that many participants relied on free programmes for their children and were excluded from activities requiring a fee or equipment. Amira took a picture of a playground (Photo 4) as one of the most visited places by her children—a place that did not have any associated costs.

Newcomer families that struggled financially due to employment difficulties could not find affordable housing in the urban centre where many programmes are delivered. This created challenges in accessing programmes and services. Afra took a photo of downtown Halifax (Photo 5) and shared in a discussion with her husband Akbar:

Afra: “Some exercises or activities were in the downtown and we are living in [Suburban area name] and we have difficulties to bring our children there.”

Akbar: “ … it would be so much easier for us regarding transportation or living close to our friends, and even regarding the schools they would be close to our children”

##### Language

All of the family participants identified language as a critical factor influencing their children’s access to programmes. Due to language differences, newcomer family members often did not know about available programmes, and when they did, encountered challenges in registering their children. Very few participants were comfortable searching for information online; instead, the majority rely on in-person interaction with service providers or through flyers sent to their homes. The registration process was one of the significant barriers Amal, Ahlam, and Amira faced. They were able to receive help from service-providers who supported them through the registration process. Amira, who was unable to secure a spot for her child in a recreational programme due to her lack of knowledge about online registration, shared a picture of a hand-written note given to her from a service provider about the online registration instructions (Photo 6).

Further, all family participants shared that the predominant use of English in the programmes influenced their children’s experiences when participating in the programmes, especially in the first few months following arrival. Families felt that language differences make it difficult for their children to make connections with others. Amal recalls her child’s frustration trying to communicate with peers at school and took a photo of her at the playground, which she called “The power of Play” (Photo 7). Amal explained that play is an important way that her child can interact with others, learn English, and build friendships.

##### Social networks

All family participants shared the critical role that their social networks play in their resettlement journey. Newcomer families who are separated from their extended families, described their post-migration social networks as a main source of financial, emotional, and informational support. For example, Habiba, who had never used a child’s car seat before arriving in Halifax, took a picture of her child in a car seat and explained that her social group helped her to find and use a car seat for her child (Photo 8). Habiba explained:

*The car seat, [we] didn’t used to have [in my country] or use it before and it’s a really useful thing and—and safe … It was very difficult to put her in a car seat when we first arrived … Our sponsors, brought and prepared the car seat before our arrival … The caring that was offered here.*

Social networks also helped families to become aware of available programmes for their children, and supported them through the registration process (as explained above). For example, many families shared that they heard about recreational activities via word of mouth from friends in their communities. Ahlam shared that after she gained access to appropriate programmes and services for her son, such as extra help for his assignments at school, she felt she was on her way to overcoming the post-migration challenges.

### Early childhood educators

**Figure 4.**
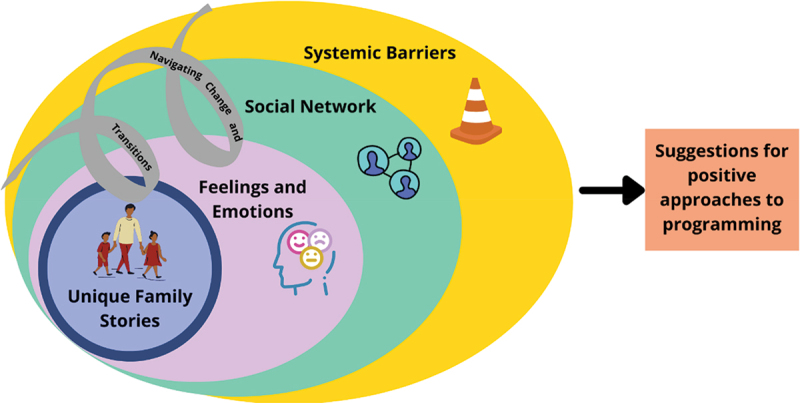
Visual representation of ECE results.

Figure 4 illustrates the four intersecting themes identified by ECEs: unique family stories; feelings and emotions; social networks and connections; and systemic barriers. The spiral represents newcomer families’ complex journey navigating change and transitions during resettlement.

#### Unique family stories

ECE participants located the *unique* needs and experiences of families at the centre of their findings, demonstrating the heterogeneity of each families’ migration experience and how it relates to access and use of services. Similar to the newcomer participants, ECE participants said that the ability of a newcomer to seek out and use programmes and services in the Canadian system is impacted by the individual family’s characteristics, experiences, and circumstances. For example, ECEs shared that the newcomers’ cultural practices, traditions, beliefs, stigma, and values around gender norms shape participants’ help-seeking behaviours. Further, newcomer ECEs believed that families’ access to programmes are also influenced by their financial stability, English-language proficiency, and awareness about available services.

Settlement journeys were also discussed as part of unique family stories that influence how newcomer families access programmes and services. The ECE participants felt that while newcomer families have some similar early migration experiences, they all respond differently. This was described by Fatima as: *“[finding a way for families to] fit themselves to make their life a little bit more comfortable.”* Fatima, Yao and Charuka described participating in cultural events as a way for them to stay connected with their family members outside of Canada. ECE participants further described how the religious, cultural practices, and traditions of newcomer families influence their access to programmes and services (Photo 9). For example, Fatima says that the newcomer families who practice fasting during Ramadan prefer to access programmes and services after they break their fast in the evening.

#### Feelings and emotions

ECE participants described their own *feelings and emotions* (and those of who they support) involved in navigating change and building relationships and trust with new programmes in Halifax,, including uncertainty, anxiety, and grief, which influenced their access to programmes and services. While newcomer families arrive with high expectations and are hopeful about rebuilding their lives in a new place, Juliana described an upheaval of emotions after migration, like a “*hurricane or tsunami*.” Charlotte further described how these feelings influenced access: *“ … uncertainty, unknowing … I think it impacts the families and how they choose the service, you know?”*

ECE participants that were also newcomers described their own complex emotions as they navigate resettlement in Canada. ECEs felt that emotions like uncertainty, fear, mistrust, isolation, and anxiety were commonly felt by newcomers. Juliana’s photo elicited discussion among ECE participants about their feelings of capable and confident newcomer children, alongside the fear that comes from sending them to a programme outside the shorter term child care delivered by the settlement agency (Photo 10). ECE participants discussed that newcomers will begin seeking information or using programmes and services when they feel safe about the system in their new environment. For example, Charlize said:

*Safety is really important because how safe do you feel if you have … if you’re really struggling with something, do you feel safe to seek help? Do you feel … who do you feel safe to go and ask for help?*

Charlize took a picture of a child holding a pot that he planted (Photo 11) representing the growth that can occur if a family feels safe and supported. Juliana, Fatima, Chruka, and Charlize agreed that it was important that programmes consider the feelings and emotions of children, and use a trauma-informed approach. The group discussed diversity among the educators at the settlement agency that they work and the familiarity of trauma-informed approaches. Chruka said:

*At the beginning they [newcomer families] want their children to be with immigrants*. *She [a newcomer mother] was telling me “that she feels safe with immigrant teachers.” I think, because they came after so many bad experiences she doesn’t want to put her children in that situation [new environment] all of a sudden. She wants to get them adjusted to the system little by little.*

Juliana also believed that using trauma-informed approaches in their programme helped them to better support newcomer families and their children. Juliana said: “ … *trauma-informed care is important. For example, the books we provide for children we check about diversity and we check all pictures and contents to not trigger some kind of feelings … we try to offer the best for children and families*.” Yao discussed other benefits from the support she received from the settlement agency as a newcomer mother:

*When I was came [sic] to Halifax, the first place like home is [settlement agency] and also the settlement advisor gave my family a lot of information … they just opened the door for me …*

#### Social networks

Like newcomer participants, ECE participants noted the importance of *social networks and connections* in helping newcomer families navigate systemic barriers and access services for their children. This importance is reflected in Fatima’s statement: *“ … if you are building connections and your network is strong in one aspect then things are going to be really easy … ”* Charlize also felt that newcomers’ informal connections with other families provides a great source of information:

*… newcomer families find … like they’re in—they’re in a bubble, you are in a bubble and it’s just the glimpses that you can see through your bubble so what … is maybe somebody on the playground says to you “do you know about this?”*

The ECE participants felt that community organizations were important in supporting the upbringing of newcomer children. Several ECEs quoted that *“it takes a village to raise a child.”* Although the participants emphasized the important role of programmes for newcomer children’s lives, they also talked about the importance of trustworthy relationships to facilitate newcomer families’ information seeking. For example, Fatima stated that though settlement agencies provided information about services, she preferred to seek information from her community and friends through WhatsApp or Facebook groups. All ECE participants said that information from trusted social circles was perceived by newcomer families as the most helpful because it was often in their preferred language. Charlotte said:

*We had a quite a few newcomer families … they were getting word of mouth referrals I guess from the friends that [sic] they had moved here before them or a lot of the time living in the same apartment building … so it seemed like a lot of that friends or family connections to help [newcomer families] find childcare in the community.*

Charlotte shared a photo of a stack of books (Photo 12). She explained that it represents that newcomer families will access information and resources once they trust service providers. ECEs further described their own interactions with families working at the childcare centre at the settlement agency where they exchanged information with newcomer families. For example, Yao shared:

*… before our class we had a little bit free talk and I know she [newcomer mother] is learning how to— how to drive cars and then wanted to get her license and the parents mentioned she failed and at that time she— she asked me “how can I register the next road test” and “I said yeah okay I help you.”*

All ECE participants agreed that the pandemic created an additional barrier that influenced newcomer families’ social network, because the restrictions limited in-person interactions, which are preferred by newcomer families. Fatima discussed the change between families and the ECEs due to the pandemic restrictions: “ *… because of COVID is the [loss of] small talk that used to happen between parents when they* would *come to pick up their children”*. Charlotte took a photo of a half-shut door that she said reflects how interactions and communication between families and ECEs have been closed off (Photo 13). Newcomer families sometimes had difficulties understanding information that was primarily delivered through emails or over the phone during the pandemic. For example, Juliana said:

*… we don’t know the language of our clients for sure and we try to use gestures, to show pictures, something to help us [communicate] and now because we use mask in the COVID time*, *pandemic time and we need to talk more with our eyes and when we are with a child in the short-term care is just we don’t have time for adaptation …*

ECE participants also described how children’s interactions have changed, such as remaining within bubbles (a term that describes a select group or cluster of people close to you, like relatives and neighbours). ECE participants discussed the importance of the environment in children’s development, especially those who have experienced trauma, when discussing a photo (14). The photo shows a bulletin board in the building where newcomer services are provided depicting a diverse group of people that was partially covered over by COVID-19 protocol sheets pinned onto the bulletin board. Although ECE participants were thankful for the pandemic safety measures they were concerned that limited family involvement was not culturally responsive to the needs of newcomer families.

#### Systemic barriers

The ECE participants stated that newcomer families experience *systemic barriers* that result in inequitable access to programmes and services. Similar to the family participants, the newcomer ECE participants identified financial, language, and technology barriers. ECE participants discussed issues related to un/underemployment, sometimes due to language differences or the lack of recognition of prior training and credentials, which can result in feelings of social exclusion as well as financial hardship. For example, Fatima shared her employment situation as a newcomer in Canada:

*… I wasn’t just an ECE back home, I was youth worker, social worker back home but I couldn't apply for that because I needed license to apply for that over here and— anywayI did come across this loan option and I was hopeful when I just had a glance at it when I got more details about it I think one thing that let me down was the interest rate again on repayment …*

Difficulty in securing a job left many of these ECE participants, who were newcomers themselves, in a difficult situation financially and emotionally during their own settlement journey.

ECE participants discussed the significant challenge of language barriers that sometimes led to a misunderstanding of information. For example, Charuka told a story about a newcomer family member who missed an appointment for her child at the hospital because she did not understand there was a follow-up visit. All of the ECE participants agreed that newcomer families with limited English proficiency experience confusion, anxiety, stress, and isolation. Referring to the Photo 14, the ECE participants pointed out that the English-only signage regarding COVID-19 protocols did not take into consideration the linguistic needs of newcomer families.

Finally, ECEs explained the language and technological difficulties that newcomer families face when filling out applications for their children’s programmes. For example, Yao recalled her own experiences as a newcomer completing a complicated subsidy application she needed to fill in when registering her child at daycare. Charlize also recounted how difficult it was for her to instruct a newcomer family member to use the online self-assessment tool for COVID-19 without an interpreter’s assistance. Fatima, whose first language is English, also shared that, upon arrival, even when she became aware of the recreational programmes for her own child through her social networks, she missed registration dates because she had a hard time navigating the program’s website.

Cultural differences faced by newcomer families were also discussed as a barrier that impacted newcomers’ experiences finding and using programmes and services for their children. Charuka made a drawing (Photo 15) which prompted a discussion about cultural differences related to “*our rights as women”*. The ECE participants discussed how gender roles and gender norms may be challenged in a new setting, including how cultural child-rearing practices in the dominant culture are being interpreted. For example, both Charuka and Charlize suggested that many newcomer families are fearful that social services will take their children away if they follow their cultural child-rearing practices that may not be acceptable in Canada. Charlize said:

*… there are cultural norms and reasons for certain things and the country or the culture where they’ve come from and it doesn’t always easily translate to a new context and culture and so it’s a yeah it’s a learning curve and that’s I think on both sides it’s a learning curve.*

Juliana took a photo of a school and playground in Nova Scotia that she describes as open without a fence perimeter (Photo 16), which she felt contradicts her perception of a safe and secure school. About her picture Juliana said:

*… this picture is my daughter school and when I arrived there, I was thinking how this school without high wall around to protect children … how the child could be safe in open space like this … as a newcomer, my first thought it is open space everywhere … [and back home] we have security guards in a reception, and we have a big brick walls [sic] around all school …*

Other ECE participants agreed with Julian’s perception of a safe school which reflects their cultural experiences. Fatima explained that her concerns about a safe school led to the decision to postpone registering her child for an early childhood programme located at the school:

*… [I] did question concept of safety here when children are playing in the open ground and roads are so close by, and I still haven’t received a satisfying answer, so I haven't send my child to big school … she goes to daycare which is a little inside.*

Lack of flexibility and adaptability of policies within the programmes have been identified as another cultural challenge faced by newcomer family members. For example, although settlement agencies adapt their programming schedule to accommodate clients who celebrate Ramadan, ECE participants did not feel that other programmes understand the importance of this and, therefore, did not respond in culturally meaningful ways.

## Discussion

Resettlement is a precarious transition for newcomers in Canada (Gelatt et al., 2014, Hernandez et al., 2009). In this study, we sought to better understand how families find and use early childhood programmes and services in Nova Scotia (Canada) that has high rates of child poverty exist, especially among immigrant children (Frank et al., 2021). Participants described the heterogeneity of family culture, religion, and ethnicity, but with common systemic barriers in relation to employment and language, which has been found in earlier studies with other newcomer families in Canada (Brown et al., 2020, Khanlou, 2010, Khanlou & Crawford, 2006, Salami et al., 2020).

This research provides further evidence of the importance of addressing parental employment and language barriers as foundational support to enable access to early childhood programmes and services. Involving a group of ECEs, most of whom were newcomers as well, provides a unique contribution alongside the findings from our family participant group. The ECEs also had overcome barriers of employment and language, and likely as a result of their professional role in supporting families articulated concepts related to the heterogeneity of family stories and the importance of trauma-informed practice.

The stories that we heard in this study are mainly from mothers’ perspectives as the majority of our participants identified as mothers with a primary caregiving responsibility. The implications of gendered roles were explicitly discussed by the ECE participants through Charuka’s drawing (Photo 15). Some ECE participants discussed the lasting impact of cultural norms related to gender, where women had less power, especially as they continued in a primary caregiving role. While cultural norms related to gender and caregiving may be changing in Canada, the COVID-19 pandemic has made it evident that there are many persisting gender inequities for women and mothers (Hillier & Greig, 2020). In this study, difficulties accessing programmes for children were believed to contribute to feelings of isolation and exclusion for mothers. Previous research has similarly found the importance of access to early learning to help newcomer caregivers, especially women, to settle by finding employment, enhancing their English language, or continuing their education (Karoly & Gonzalez, 2011, Leung et al., 2019).

As captured in their photovoice stories, newcomers demonstrate their capacity to change their life trajectory, to adapt to new cultures, and resettle in a new country. At the same time, systemic barriers provide limited options to support newcomers when entering Canada. There continues to be significant gaps in services as it pertains to culturally relevant early years pedagogies, social supports, and universal design related to education and employment (Choi et al., 2014, Salami et al., 2020). The findings of this study demonstrate that successful resettlement requires a deeper understanding of the complex and unique needs of newcomer families (Salami et al., 2022,)). More consideration of previous credentials and work experiences of the newcomer families’ is needed, alongside of accessible and effective ways to enhance English language, and enhancing community and educational supports and services that address the inequities that many newcomers experience (Karoly & Gonzalez, 2011, Salami et al., 2019, Walker & Zuberi, 2020).

Similar to other studies, the importance of collaboration with families was mentioned by ECE participants, which was accomplished by welcoming families into programming and demonstrating respect for individual differences and needs (Ansion & Merali, 2018, Browne et al., 2018, Klassen et al., 2012, Wahoush, 2009). Through a poststructural lens, we see the issue of voice emerge and the importance of embracing multiculturalism and intersectionality in our programmes and services. As hooks (1994) explains “to hear each other … is an exercise in recognition” (p. 41). ECEs and family participants all spoke about the importance of play as a universal language for children, that supports child social and emotional development and their resettlement journey. Play is a multifaceted concept, and a recognized right of a child (Gosso & Almeida Carvalho, 2013; Unicef, n.d..), with different theoretical foundations that inform its relationship to learning and development (Sutton-Smith, 2008). Importantly, social and cultural constructs must also be recognized as significant aspects that influence perceptions of play (Gosso & Almeida Carvalho, 2013, Yahya & Wood, 2017), which participants in this study suggested was not commonly understood among programmes offered in the community.

Participants highlighted the importance of culturally-responsive practices in programmes and services that newcomer children access. For example, trauma-informed approaches in programmes were specifically named by the ECE participants who believed that newcomer families, particularly those of refugee backgrounds arrive with sometimes adverse pre-migration experiences and continue to experience traumatic experiences during settlement (e.g., bullying, racism). Trauma-informed practices have been found to support immigrant children who experience social, emotional, educational, and behavioural disparities due to pre-and post-migration traumatic experiences (Jacobson, 2021, Kia-Keating & Ellis, 2007, Stewart, 2012, Walker & Zuberi, 2020). Trauma-informed practice encourages creating a safe and welcoming environment for immigrant children where their pre-and post-migration experiences are acknowledged, and inter-cultural interactions are emphasized and supported (Jacobson, 2021, Kia-Keating & Ellis, 2007, Stewart, 2012, Walker & Zuberi, 2020).

This study corroborates earlier literature suggesting the importance of post-migration social networks as a main source of financial, emotional, and informational support. As hooks (1994) reminds us “we must build ‘community’ in order to create a climate of openness” (p. 40) and for the newcomers their capacity to become part of community was a reliance on building social networks. Other studies also emphasize the role of social support in immigrant families’ settlement journeys, especially in helping newcomer families become aware of programmes and services available for their children (Brown et al., 2020, Khanlou, 2010, Stirling Cameron et al., 2022)). For example, Khanlou (2010) suggests that social networking among ethnic communities improves immigrants’ mental health by enhancing their abilities to navigate post-migration challenges like finding employment. Syrian mothers in Nova Scotia also stress that their new social groups improve their mental well-being after giving birth as they become like their “chosen family,” providing support and taking care of their children (Stirling Cameron et al., 2022, p. 105). Brigham et al. (2018) found that for young immigrant and refugee women in Halifax (Nova Scotia), social networks have a positive effect on their daily lives (e.g., helping them to feel accepted in their new environment, giving them a sense of belonging and well-being). The participants in Brigham et al.’s study expressed that new friendships, in particular, “offer security, hope and comfort as well as critical supports in their challenges to navigate, adapt, adjust and settle in diverse environments” (p. 11).

### Strengths and limitations of the study

This study used a feminist post-structural lens through the use of photovoice to prioritize the voices of newcomer families that experience marginalization and are often excluded from traditional research (Wang & Burris, 1994). The number of participants allowed for sufficient data for the planned depth of analysis while also ensuring meaningful participation (Wang, 2006). Our inclusion of perspective of both newcomer families and service providers (ECEs) is a strength as it deepened our understanding of access and use of services for young children. ECE participants offered unique perspectives as most were able to reflect upon their own journey, as well as their close experiences working to support newcomer families and children. In this way, the ECEs were able to reflect upon their experiences in relation to others whereas family participants mostly recounted their own personal stories. Further, the ECE group was able to reflect on both the professional and personal experience within the newcomer population, which was not an intentional purpose but a result that enriched the findings. Photovoice offered a creative and meaningful engagement opportunity for participants, allowing further expression of ideas beyond words. Participants reflected symbolism and metaphors in their photos (e.g., flowers blooming through a storm), which permitted meaning making that may not have been so easily expressed in words.

We also assert that conducting the Photovoice process virtually was valuable, especially during the pandemic, as it allowed connection to newcomers at a time of isolation. Our inclusion of interpreters ensured that newcomer families with limited English proficiency were able to participate. However, we do acknowledge the potential limitation of interpreters given nuances of expressions and language. Further, an additional limit of our study was more limited dialogue between participants given the online format and that our family participants chose to take part with a close family member or on their own. We built relationships using engaging questions, sharing our own stories in relation to difficulties in accessing services, and brought the family participants together at a final session to discuss the combined result but the virtual format made it more difficult for deeper rich dialogue between participants.

### Implications

The results from this research were shared locally to inform changes to programmes and services, including addressing systemic barriers of employment and language, leveraging existing social networks, and play-based, trauma-informed approaches. Participants decided to shared their photos at an outdoor public photo exhibit. The exhibit later travelled to other public libraries and spaces. The research team also delivered a number of presentations to government departments. The results of this research may provide inspiration to further amplify the voices of newcomer families to challenge monocultural perspectives and destabilize universal truths. Additional research is recommended to further refine recommendations for a virtual-based photovoice approach given its potential to expand the reach for participation for populations that are marginalized, such as those located in rural or isolated areas, while maintaining the rich dialogue and group participant-led analysis between participants that is a common focus in the methodology. Participatory research is also needed to capture the first-voice experiences of newcomer children, which was beyond the scope of this study but a critical area for future research.

## Conclusions

This research aimed to gain insight into how newcomer families in Nova Scotia access programmes and services for their children. The photovoice methodology allowed newcomer families and ECEs to engage in research and illustrate their experiences using photos instead of relying solely on oral language. Further, the method of conducting this research virtually was responsive to the unique needs of newcomer families during the pandemic and enhanced participation. Future in-person research would help to further our understanding of the experiences in early childhood programmes from the perspective of families and the children, including how trauma-informed practices are adopted and to further the complexity of gender relations in caregiving. This research highlights the importance of culturally responsive programmes where the unique needs of newcomer children and their families are considered and the need to address the systematic barriers, such as access to employment and education, that impact newcomer families. We found that social networks support family navigation and enhance families’ access to programmes and services, which emphasizes their important role in settlement journeys. Future research needs to prioritize the voices of families that experience marginalization, and where possible include the first-voice perspective of children who are directly impacted by programmes and services.

## Geolocation information

This research was conducted in Halifax, Nova Scotia, Canada.

## References

[cit0001] Ansion, M., & Merali, N. (2018). Latino immigrant parents’ experiences raising young children in the absence of extended family networks in Canada: Implications for counselling. *Counselling Psychology Quarterly*, 31(4), 408–17. 10.1080/09515070.2017.1324760

[cit0002] Azzarito, L. (2012). Photography as a pedagogical tool for shedding light on ‘bodies-at-risk’ in physical culture. *Visual Studies*, 27(3), 295–309. aph. 10.1080/1472586X.2012.717746

[cit0003] Azzarito, L., Solmon, M. A., & Harrison, L. (2006). “.If I had a choice, I would.” a feminist poststructuralist perspective on girls in physical Education. *Research Quarterly for Exercise and Sport*, 77(2), 222–239. 10.1080/02701367.2006.10599356 16898278

[cit0004] Barrett, M. J. (2010). Making (some) sense of feminist poststructuralism in Environmental Education research and practice. *Canadian Journal of Environmental Education*, 10(1), 79–93.

[cit0005] Benjamen, J., Girard, V., Jamani, S., Magwood, O., Holland, T., Sharfuddin, N., & Pottie, K. (2021). Access to refugee and Migrant mental Health care services during the first six months of the COVID-19 pandemic: A Canadian refugee clinician Survey. *International Journal of Environmental Research and Public Health*, 18(10), 5266. 10.3390/ijerph18105266 34063442 PMC8156129

[cit0006] Brigham, S. (2015). Mothering has no borders: The transnational kinship networks of undocumented Jamaican domestic workers in Canada. In R. Cohen, & G. Man (Eds.), *Engendering transnational voices: Studies in family, work, and identity* (pp. 135–153). Wilfrid Laurier Press.

[cit0007] Brigham, S., Baillie-Abidi, C., & Calatayud, S. (2018). Migrant women learning & teaching through participatory photography. *Canadian Journal for the Study of Adult Education*, 30(2), Article 2. https://cjsae.library.dal.ca/index.php/cjsae/article/view/5433

[cit0008] Brigham, S. M., Baillie Abidi, C., & Zhang, Y. (2018). What participatory photography can tell us about immigrant and refugee women’s learning in Atlantic Canada. *International Journal of Lifelong Education*, 37(2), 234–254. 10.1080/02601370.2017.1422044

[cit0009] Brigham, S. M., & Kharbach, M. (2020). Ethical issues in a participatory photography research project involving Youth with refugee experience. In S. Dodd (Ed.), *Ethics and integrity in visual research methods* (Vol. 5, pp. 153–170). Emerald Publishing Limited. 10.1108/S2398-601820200000005014

[cit0010] Brosinsky, L., Georgis, R., Gokiert, R., Mejia, T., & Kirova, A. (2018). RAISED between cultures: New resources for working with children of immigrant or refugee background. *Childhood Education*, 94(2), 18–27. 10.1080/00094056.2018.1451686

[cit0011] Brotman, S., Koehn, S., Ferrer, I., Koehn, S. D., Badger, M., Sohng, K., Lang, A., Li, K. N., & Bukhari, S. (2017). The lived experiences of aging immigrants a narrative-photovoice project 2014-2017. *Innovation in Aging*, 1(suppl_1), 645–645. 10.1093/geroni/igx004.2285

[cit0012] Browne, D. T., Kumar, A., Puente-Duran, S., Georgiades, K., Leckie, G., & Jenkins, J. (2017). Emotional problems among recent immigrants and parenting status: Findings from a national longitudinal study of immigrants in Canada. *PLoS ONE*, 12(4), e0175023. 10.1371/journal.pone.0175023 28376118 PMC5380348

[cit0013] Browne, D. T., Wade, M., Prime, H., & Jenkins, J. M. (2018). School readiness amongst urban Canadian families: Risk profiles and family mediation. *Journal of Educational Psychology*, 110(1), 133–146. 10.1037/edu0000202

[cit0014] Brown, A., McIsaac, J.-L. D., Reddington, S., Hill, T., Brigham, S., Spencer, R., & Mandrona, A. (2020). Newcomer families’ experiences with programs and services to support early childhood development in Canada: A scoping review. *Journal of Childhood, Education & Society*, 1(2), 182–215. 10.37291/2717638X.20201249

[cit0015] Castleden, H., Garvin, T., & First Nation, H. (2008). Modifying photovoice for community-based participatory indigenous research. *Social Science & Medicine*, 66(6), 1393–1405. 10.1016/j.socscimed.2007.11.030 18191883

[cit0016] Choi, J., Kushner, K., Mill, J., & Lai, D. (2014). The experience of Korean immigrant women adjusting to Canadian Society. *Journal of Cross-Cultural Gerontology*, 29(3), 277–297. aph. 10.1007/s10823-014-9235-8 25096026

[cit0017] Drolet, J., Robertson, J., Multani, P., Robinson, W., & Wroz, M. (2008). Settlement experiences in a small city: Kamloops, British Columbia. *Small Cities Imprint*, 1(1), Article 1. https://smallcities.tru.ca/index.php/cura/article/view/6

[cit0018] Etowa, J., Sano, Y., Hyman, I., Dabone, C., Mbagwu, I., Ghose, B., Osman, M., & Mohamoud, H. (2021). Difficulties accessing health care services during the COVID-19 pandemic in Canada: Examining the intersectionality between immigrant status and visible minority status. *International Journal for Equity in Health*, 20(1), 255. 10.1186/s12939-021-01593-1 34915891 PMC8674863

[cit0019] Fassetta, G. (2016). Using photography in research with young migrants: Addressing questions of visibility, movement and personal spaces. *Children’s Geographies*, 14(6), 701–715. 10.1080/14733285.2016.1190811

[cit0020] Frank, L., Fisher, L., & Saulnier, C. (2021). 2021 report card on child and family poverty in Nova Scotia (p. 62). https://policyalternatives.ca/publications/reports/2021-report-card-child-and-family-poverty-nova-scotia

[cit0021] Gelatt, J., Adams, G., & Huerta, S. (2014). Supporting immigrant families’ access to Prekindergarten. *The Urban Institute*, 37, 6–8.

[cit0022] Gosso, Y., & Almeida Carvalho, A. M. (2013). Play and cultural context. *Encyclopedia on Early CHildhood Development*. https://www.child-encyclopedia.com/pdf/expert/play/according-experts/play-and-cultural-context

[cit0023] Government of Canada, S. C. (2017, October 25). *The Daily — Immigration and Ethnocultural Diversity: Key Results from the 2016 Census*. https://www150.statcan.gc.ca/n1/daily-quotidien/171025/dq171025b-eng.htm

[cit0024] Guruge, S., Lamaj, P., Lee, C., Ronquillo, C. E., Sidani, S., Leung, E., Ssawe, A., Altenberg, J., Amanzai, H., & Morrison, L. (2021). COVID-19 restrictions: Experiences of immigrant parents in Toronto. *AIMS Public Health*, 8(1), 172–185. 10.3934/publichealth.2021013 33575415 PMC7870386

[cit0025] Haque, N., & Eng, B. (2011). Tackling inequity through a photovoice project on the social determinants of health: Translating photovoice evidence to community action. *Global Health Promotion*, 18(1), 16–19. 10.1177/1757975910393165 21721294

[cit0026] Hernandez, D. J., Takanishi, R., & Marotz, K. G. (2009). Life circumstances and public policies for young children in immigrant families. *Early Childhood Research Quarterly*, 24(4), 487–501. 10.1016/j.ecresq.2009.09.003

[cit0027] Hesse-Biber, S. N. (2012). *Handbook of feminist research: Theory and praxis* (2nd ed.). SAGE. 10.4135/9781483384740.n1

[cit0028] Hillier, K. M., & Greig, C. J. (2020, September 13). Motherhood and mothering during COVID-19: A gendered intersectional analysis of caregiving during the global pandemic within a Canadian context. - *JourMs -*. https://jourms.org/motherhood-and-mothering-during-covid-19-gendered-intersectional-analysis-of-caregiving-during-the-global-pandemic-within-a-canadian-context/

[cit0029] Jacobson, M. R. (2021). An exploratory analysis of the necessity and utility of trauma-informed practices in education. *Preventing School Failure: Alternative Education for Children & Youth*, 65(2), 124–134. 10.1080/1045988X.2020.1848776

[cit0030] Karoly, L. A., & Gonzalez, G. C. (2011). Early care and education for children in immigrant families. *The Future of Children*, 21(1), 71–101. 10.1353/foc.2011.0005 21465856

[cit0031] Khanlou, N. (2010). Migrant mental health in Canada. *Canadian Issues*, 9–16 https://acs-aec.ca/wp-content/uploads/2019/05/CITC-2010-Summer-Ete-L-1.pdf

[cit0032] Khanlou, N., & Crawford, C. (2006). Post-migratory experiences of newcomer female youth: Self-esteem and identity development. *Journal of Immigrant & Minority Health*, 8(1), 45–56. 10.1007/s10903-006-6341-x 19834999

[cit0033] Kia-Keating, M., & Ellis, B. H. (2007). Belonging and connection to school in resettlement: Young refugees, school belonging, and psychosocial adjustment. *Clinical Child Psychology and Psychiatry*, 12(1), 29–43. 10.1177/1359104507071052 17375808

[cit0034] Klassen, A. F., Gulati, S., Watt, L., Banerjee, A. T., Sung, L., Klaassen, R. J., Dix, D., Poureslami, I. M., & Shaw, N. (2012). Immigrant to Canada, newcomer to childhood cancer: A qualitative study of challenges faced by immigrant parents. *Psycho-Oncology*, 21(5), 558–562. aph. 10.1002/pon.1963 21425390

[cit0035] LaRochelle-Côté, S., & Uppal, S. (n.d.). The social and economic concerns of immigrants during the COVID-19 pandemic. 6.

[cit0036] Lather, P. (1997). Drawing the line at angels: Working the ruins of feminist ethnography. *International Journal of Qualitative Studies in Education*, 10(3), 285–304. 10.1080/095183997237124

[cit0037] Leung, V. W. Y., Zhu, Y., Peng, H.-Y., & Tsang, A. K. T. (2019). Chinese immigrant mothers negotiating family and career: Intersectionality and the role of social support. *The British Journal of Social Work*, 49(3), 742–761. 10.1093/bjsw/bcy081

[cit0038] Massing, C. (2021). *Introduction to early childhood education and care: An intercultural perspective*. Brush Education.

[cit0039] McHugh, M. P., & Margie. (2014, May 30). *Immigrant Parents and Early Childhood Programs: Addressing Barriers of Literacy, Culture, and Systems Knowledge*. Migrationpolicy.Org. https://www.migrationpolicy.org/research/immigrant-parents-early-childhood-programs-barriers

[cit0040] Miller, Meghan. (2021). New In the Wheat City: a photovoice exploration of new residents’ relationship with community and place. https://viurrspace.ca/bitstream/handle/10613/23525/Miller_royalroads_1313O_10731.pdf?sequence=1&isAllowed=y

[cit0041] Nova Scotia, (2021, November 25). *Department of Labour, Skills and Immigration*. Communications Nova Scotia. https://beta.novascotia.ca/government/labour-skills-and-immigration

[cit0042] Nova Scotia. (2023). *Learn About Early Learning and Child Care in NS | Education and Early Childhood Development*. https://www.ednet.ns.ca/ece/elcc-in-ns

[cit0043] Pacini-Ketchabaw, V., & Bernhard, J. K. (2018). 5 revisioning multiculturalism in early childhood Education. In *5. Revisioning multiculturalism in early childhood education* (pp. 159–181) University of Toronto Press. 10.3138/9781442662032-008

[cit0044] Perlman, M., & Fletcher, B. A. (2012). Hellos and how are yous: Predictors and correlates of communication between staff and families during morning drop-off in child care centers. *Early Education and Development*, 23(4), 539–557. 10.1080/10409289.2010.548766

[cit0045] Pierre, E., Pillow, W., & Pierre, E. S. (1999). *Working the Ruins: Feminist Poststructural Theory and Methods in Education*. Taylor & Francis Group. http://ebookcentral.proquest.com/lib/msvu/detail.action?docID=170226

[cit0046] Rania, N., Migliorini, L., Rebora, S., & Cardinali, P. (2015). Photovoice and interpretation of pictures in a group discussion: A community Psychology approach. *Qualitative Research in Psychology*, 12(4), 382–396. 10.1080/14780887.2015.1019597

[cit0047] Reynolds, B., & Duff, K. (2016). Families’ perceptions of early childhood educators’ fostering conversations and connections by sharing children’s learning through pedagogical documentation. *Education 3-13*, 44(1), 93–100. 10.1080/03004279.2015.1092457

[cit0048] Robertson, Z., Gifford, S., McMichael, C., & Correa-Velez, I. (2016). Through their eyes: Seeing experiences of settlement in photographs taken by refugee background youth in Melbourne, Australia. *Visual Studies*, 31(1), 34–49. 10.1080/1472586X.2015.1128845

[cit0049] Salami, B., Alaazi, D. A., Ibrahim, S., Yohani, S., Scott, S. D., Vallianatos, H., Urichuk, L., & Islam, B. (2022). African immigrant parents’ perspectives on the factors influencing their children’s mental Health. *Journal of Child and Family Studies*, 31(1), 142–154. 10.1007/s10826-021-02130-y

[cit0050] Salami, B., Alaazi, D. A., Okeke‐Ihejirika, P., Yohani, S., Vallianatos, H., Tetreault, B., & Nsaliwa, C. (2020). Parenting challenges of African immigrants in Alberta, Canada. *Child & Family Social Work*, 25(S1), 126–134. 10.1111/cfs.12725

[cit0051] Salami, B., Salma, J., & Hegadoren, K. (2019). Access and utilization of mental health services for immigrants and refugees: Perspectives of immigrant service providers. *International Journal of Mental Health Nursing*, 28(1), 152–161. 10.1111/inm.12512 29984880

[cit0052] Sethi, B. (2016). Using the eye of the camera to bare racism: A photovoice project. *Aotearoa New Zealand Social Work*, 28(4), 17–28. 10.11157/anzswj-vol28iss4id294

[cit0053] Stewart, J. (2012). Transforming schools and strengthening leadership to support the educational and psychosocial needs of war-affected children living in Canada. *Diaspora, Indigenous, and Minority Education*, 6(3), 172–189. eric. 10.1080/15595692.2012.691136

[cit0054] Stirling Cameron, E., Aston, M., Ramos, H., Kuri, M., & Jackson, L. (2022). The postnatal experiences of resettled Syrian refugee women: Access to healthcare and social support in Nova Scotia, Canada. *Midwifery*, 104, 103171. 10.1016/j.midw.2021.103171 34736018

[cit0055] Stirling Cameron, E., Ramos, H., Aston, M., Kuri, M., & Jackson, L. (2021). “COVID affected us all:” the birth and postnatal health experiences of resettled Syrian refugee women during COVID-19 in Canada. *Reproductive Health*, 18(1), 256. 10.1186/s12978-021-01309-2 34952615 PMC8709538

[cit0056] Strack, R. W., Magill, C., & Mcdonagh, K. (2004). Engaging youth through photovoice. *Health Promotion Practice*, 5(1), 49. 10.1177/1524839903258015 14965435

[cit0057] Sutherland, C., & Cheng, Y. (2009). Participatory-action research with (Im)migrant women in two small Canadian cities: Using photovoice in Kingston and Peterborough, Ontario. *Journal of Immigrant & Refugee Studies*, 7(3), 290–307. 10.1080/15562940903150089

[cit0058] Sutton-Smith, B. (2008). Play Theory: A personal journey and new Thoughts. *American Journal of Play*, 1(1), 80–123.

[cit0059] Thevenot. (2021). *Immigration Helps Nova Scotia’s Population Surpass 1 Million | Canada Immigration News*. https://www.cicnews.com/2021/12/immigration-helps-nova-scotias-population-surpass-1-million-1220434.html

[cit0060] Unicef. (n.d.). *The Convention on the Rights of the Child: The Child-Friendly Version*. https://www.unicef.org/sop/convention-rights-child-child-friendly-version

[cit0061] Vesely, C. K., Ewaida, M., & Kearney, K. B. (2013). Capitalizing on early childhood Education: Low-income immigrant mothers’ use of early childhood Education to build Human, social, and navigational capital. *Early Education and Development*, 24(5), 744–765. 10.1080/10409289.2012.725382

[cit0062] Wahoush, E. O. (2009). Equitable health-care access: The experiences of refugee and refugee claimant mothers with an ill preschooler. *Canadian Journal of Nursing Research*, 41(3), 186–206. 19831061

[cit0063] Walker, J., & Zuberi, D. (2020). School-aged Syrian refugees resettling in Canada: Mitigating the effect of pre-migration trauma and post-migration discrimination on academic achievement and psychological well-being. *Journal of International Migration & Integration*, 21(2), 397–411. 10.1007/s12134-019-00665-0

[cit0064] Wang, C. C. (2006). Youth participation in photovoice as a strategy for community change. *Journal of Community Practice*, 14(1–2), 147–161. 10.1300/J125v14n01_09

[cit0065] Wang, C., & Burris, M. A. (1994). Empowerment through photo novella: Portraits of participation. *Health Education Quarterly*, 21(2), 171–186. 10.1177/109019819402100204 8021146

[cit0066] Wang, C., & Burris, M. A. (1997). Photovoice: Concept, methodology, and use for participatory needs assessment. *Health Education & Behavior*, 24(3), 369–387. 10.1177/109019819702400309 9158980

[cit0067] Wang, S., Moss, J. R., & Hiller, J. E. (2006). Applicability and transferability of interventions in evidence-based public health. *Health Promotion International*, 21(1), 76–83. 10.1093/heapro/dai025 16249192

[cit0068] Wang, C. C., Yi, W. K., Tao, Z. W., & Carovano, K. (1998). Photovoice as a participatory Health Promotion strategy. *Health Promotion International*, 13(1), 75–86. 10.1093/heapro/13.1.75

[cit0069] Weedon, C. (1987). *Feminist Practice and Poststructuralist Theory*. BBlackwell.

[cit0070] Yahya, R., & Wood, E. A. (2017). Play as third space between home and school: Bridging cultural discourses. *Journal of Early Childhood Research*, 15(3), 305–322. 10.1177/1476718X15616833 28867965 PMC5555448

